# Investigating the Presence of Rotavirus in Wastewater Samples of Bhopal Region, India, by Utilizing Droplet Digital Polymerase Chain Reaction

**DOI:** 10.7759/cureus.58882

**Published:** 2024-04-24

**Authors:** Ram K Nema, Ashutosh K Singh, Juhi Nagar, Bhavna Prajapati, Mudra Sikenis, Surya Singh, Vishal Diwan, Pushpendra Singh, Rajnarayan Tiwari, Pradyumna K Mishra

**Affiliations:** 1 Division of Environmental Biotechnology, Genetics, and Molecular Biology, Indian Council of Medical Research (ICMR) - National Institute for Research in Environmental Health, Bhopal, IND; 2 Division of Environmental Monitoring and Exposure Assessment (Water & Soil), Indian Council of Medical Research (ICMR) - National Institute for Research in Environmental Health, Bhopal, IND; 3 Division of Microbial Genomics, Indian Council of Medical Research (ICMR) - National Institute for Research in Tribal Health, Jabalpur, IND; 4 Division of Epidemiology and Public Health, Indian Council of Medical Research (ICMR) - National Institute for Research in Environmental Health, Bhopal, IND

**Keywords:** environmental surveillance, gastroenteritis, wastewater-based epidemiology, droplet digital pcr, rotavirus

## Abstract

Introduction: Rotavirus-induced viral gastroenteritis outbreaks result in over two million hospitalizations globally yearly. Wastewater-based epidemiology (WBE) has emerged as a crucial tool for detecting and monitoring viral outbreaks. The adoption of WBE has been instrumental in the early detection and surveillance of such viral outbreaks, providing a non-invasive method to assess public health.

Objective: This study aims to utilize droplet digital polymerase chain reaction (ddPCR) technology to detect and quantify Rotavirus in wastewater samples collected from the Bhopal region of India, thereby contributing to the understanding and management of viral gastroenteritis outbreaks through environmental surveillance.

Methods: In this study, we used ddPCR to detect and quantify Rotavirus in wastewater samples collected from the Bhopal region of India. We monitored its viral presence in municipal sewage treatment plants bi-weekly using an advanced ddPCR assay. Targeting the rotavirus non-structural protein 3 (NSP-3) region with custom primers and TaqMan probes, we detected virus concentration employing polyethylene glycol (PEG). Following RNA isolation, complementary DNA (cDNA) synthesis, and ddPCR analysis, our novel method eliminated standard curve dependence, propelling virus research and treatment forward.

Results: Out of the 42 samples collected, a 16.60% positivity rate was observed, indicating a moderate presence of Rotavirus in Bhopal. The wastewater treatment plants (WWTP) attached to a hospital exhibited a 42.85% positivity rate, indicating the need for targeted monitoring. Leveraging ddPCR, precise quantification of rotavirus concentrations (ranging from 0.75 to 28.9 copies/µL) facilitated understanding and supported effective remediation.

Conclusions: This study emphasizes the importance of vigilant wastewater surveillance, especially in WWTPs with higher rotavirus prevalence. The significance of ddPCR in comparison to conventional and real-time PCR lies in its superior sensitivity and specificity in detecting and quantifying positive samples. Furthermore, it can identify positive samples even in the smallest quantities without the need for a standard curve to evaluate. This makes ddPCR a valuable tool for accurate and precise detection and quantification of samples.

## Introduction

Rotaviruses stand as the primary etiological agents behind severe and dehydrating diarrhea in children below the age of five globally, causing a substantial health burden. Rotavirus infection is a significant global health concern, accounting for more than 25 million outpatient visits and two million hospitalizations annually [1]. In India, it causes approximately 11 million cases of acute gastroenteritis in children under the age of five every year [2]. Recent research has revealed the gravity of the situation, with estimates indicating that Rotavirus-associated diarrhea is responsible for the loss of 122,000-153,000 young lives in India annually [3]. It is a global health concern that affects both humans and animals. It is crucial to have a comprehensive understanding of its epidemiology, clinical manifestations, and preventive measures. The zoonotic potential of Rotavirus blurs the lines of transmission, emphasizing the need for a holistic approach to prevention and control strategies [4]. This zoonotic potential holds significant implications for both animal and human health, blurring the lines of transmission and necessitating a comprehensive approach to prevention and control strategies.

Rotaviruses are named after their wheel-like appearance. They belong to the Rotavirus genus and are composed of nine species designated 'A' through 'J' [5]. Rotavirus A is the primary cause of infection in humans. It has two types of proteins, VP7 and VP4, which determine the G and P serotypes, respectively [6]. The genome of the virus is comprised of 11 segments, each of which encodes a unique protein, except for the 11th segment, which encodes two different non-structural proteins, namely, NSP5 and NSP6, in specific strains [7]. These segments vary in size from 667 to 3,302 nucleotides [8].

Rotavirus is responsible for 40.78% of diarrheal illness cases in Southeast Asia between 2008 and 2018 [9]. The prevalence of this virus in Asia varies seasonally and is influenced by regional climate and environmental factors. Studies conducted in Southeast Asia and Thailand have shown that the prevalence of rotavirus infections is higher during cooler, drier months, typically peaking in winter between October and February [9]. In South Asia, especially in Bangladesh, the number of rainy days and temperature range significantly affect the rotavirus transmission, with peak activity in colder months [10]. In India, the prevalence of Rotavirus varies geographically, with a higher incidence in winter and low humidity in the northern regions [11]. Despite regional differences, these studies collectively indicate the impact of environmental and climatic factors on the seasonal patterns of rotavirus infections in Asia. In North America, Rotavirus induces seasonal peaks of gastroenteritis, typically occurring from November to May annually. The onset of activity initiates in the Southwest United States and subsequently spreads to the Northeast, as evidenced by previous studies [12]. Although Rotavirus is detected year-round in almost every country, distinctive seasonal patterns underscore the relevance of geographical variations in rotavirus prevalence during the dry months. It highlights the importance of targeted surveillance and intervention strategies, particularly during the specified period every year.

Compelling evidence supports Rotavirus as a waterborne pathogen, capable of maintaining infectivity in aqueous environments for several days, leading to waterborne transmission and contributing to numerous outbreaks [13]. The well-established person-to-person transmission occurs through the fecal-oral route [14], with developing countries additionally susceptible to fecally contaminated water transmission [13]. While respiratory spread through small particle aerosol has been suspected, it remains unproven in humans [15]. These transmission pathways underscore the multifaceted nature of rotavirus spread, warranting diverse preventive strategies. Disinfection techniques have been highly effective in treating waterborne viruses. Ultraviolet (UV) irradiation impacts genomic material by forming pyrimidine dimers that destroy nucleic acids or through photoproducts that lead to direct photolysis of photosensitive viral components [16]. The ozone-based inactivation of viruses is mainly associated with the direct oxidation of capsid proteins through the formation of free radicals [16]. In contrast, chlorine or chlorine dioxide disinfection inactivates viruses by causing genetic or protein damage [17].

Waterborne transmission is particularly impactful in developing countries, where fecally contaminated water is a common issue [18]. Direct person-to-person spread via the fecal-oral route remains the primary transmission pathway, but the potential for respiratory spread through aerosolized particles, although suspected, is not yet conclusively proven in humans [18]. The complex transmission dynamics of Rotavirus necessitate multifaceted preventive measures.

Methods commonly used for the detection of Rotavirus include electron microscopy, gel electrophoresis, antigen detection assays, RT-PCR, and virus isolation [19]. In recent years, the evolution of rotavirus diagnostics has shifted from traditional methods, such as enzyme immunoassay (EIA) and electron microscopy, to advanced molecular and immunological techniques. The introduction of reverse transcription-polymerase chain reaction (RT-PCR), real-time polymerase chain reaction (PCR), and immunochromatographic assays has significantly enhanced sensitivity and specificity, allowing for precise and rapid detection of rotavirus infections [20]. These innovations improve diagnostic accuracy and hold promise for point-of-care applications, particularly in resource-limited settings. However, there is a need for a comprehensive approach to improve detection methods and overcome the sensitivity issues in current diagnostic tests [21]. It is also essential to explore preventive strategies based on detecting viruses in wastewater to manage outbreaks effectively. Wastewater-based epidemiology (WBE), a recent development, serves as an early monitoring system for the (re-)emergence of various pathogens and outbreak identification without relying on patient data [22]. Researchers uncover insights into its epidemiology, evolution, and ecological dynamics by analyzing genetic sequences in diverse rotavirus strains. Surveillance of sewage water can serve as an early indicator of virus occurrence among a given local population of a region by screening WWTP. This investigation is pivotal in developing more effective strategies to prevent or manage rotavirus infections within the community ddPCR provides absolute quantification over conventional PCR methods with better accuracy and sensitivity [23]. The main objectives of our study are to assess the prevalence of Rotavirus in wastewater samples from Bhopal and evaluate the effectiveness of ddPCR in detecting and quantifying the virus.

## Materials and methods

Waste water sample collection: In this cross-sectional study, sewage samples were collected from five different WWTPs in Bhopal, Madhya Pradesh, India, from April to July 2023. These inlet samples from WWTPs were collected systematically on a fortnightly basis, each 50 mL in volume. The sampling sites covered diverse locations in Bhopal. To ensure the integrity of viral nucleic acid for subsequent analysis, samples were immediately subjected to RNA isolation or refrigerated at 4°C and processed within 24 hours.

Virus concentration using PEG precipitation method: The PEG precipitation method was employed to concentrate the virus present in the sample. Initially, 50 mL of sewage samples were centrifuged (Eppendorf 5910Ri; Eppendorf Corporate, Leipzig, Germany) at 4,500 x g for 30 minutes and filtered using 0.22 µm filters. The supernatant's pH was then adjusted to 7.0-7.5, and 10% PEG 8,000 (Himedia GRM7402-500G; HiMedia Laboratories, LLC, Kennett Square, PA) and NaCl (Sigma MB3M73004; Sigma-Aldrich, Inc., St. Louis, MO) were added to achieve a final concentration of 0.3 molar. The sample was left to incubate overnight at 4°C and then centrifuged at 4,500 x g for 30 minutes to obtain pellets. These were resuspended in 500 µL of phosphate buffer saline (PBS) (Himedia ML116-500ML; HiMedia Laboratories, LLC) to form a virus concentrate that could be processed immediately or stored at −80°C for future nucleic acid extraction [24].

RNA extraction: The virus concentrate's RNA extraction process was carried out using the AllPrep Power Viral DNA/RNA Kit provided by Qiagen, Germany (Cat. No 28000-50), following the manufacturer's instructions. The elution buffer was used to elute the extracted RNA into a volume of 100 µL, which was then stored in 10 µL aliquots at a temperature of -80°C until use.

cDNA synthesis: The GoScript™ reverse transcription system (Cat. No. A5000) manufactured by Promega Corporation (Madison, WI) was employed as per the manufacturer's protocol, and the resulting cDNA was stored at −80°C for future use.

ddPCR assay: The NSP-3 regions of the Rotavirus genotype were targeted using primers and TaqMan probes, which were selected based on their high specificity as validated through the Basic Local Alignment Search Tool (BLAST) program from NCBI. The TaqMan probe, which employed 5'-FAM as a fluorescent reporter dye and 3'-BHQ-1 as a quencher, provided accurate signal detection during the assay. The sequences of the primers and probes were derived from a previously published article [25]. For the ddPCR reaction setup, 2 µL (50 ng) of cDNA was combined with a reaction mixture containing ddPCR supermix for probes, forward and reverse primers, and the TaqMan probe. An automatic droplet generator, QX200 AutoDG (Bio-Rad Laboratories, Inc., Hercules, CA), was utilized to generate droplets in a 96-well polypropylene plate. After droplet generation, PCR cycling was performed, consisting of initial denaturation, followed by 45 cycles of denaturation (95°C for 15 seconds), annealing, and extension (55°C for 30 seconds), followed by a final denaturation step (55°C for seven minutes). After PCR cycling was completed, the droplet-containing plate was transferred to a QX200 droplet reader for analysis. The QuantaSoft (pro version 1.2; Bio-Rad Laboratories, Inc.) analysis software was used for data analysis to distinguish positive from negative droplets based on fluorescence amplitude, thereby identifying positive droplets containing the amplification products. This Poisson statistics method provided accurate quantification and high-resolution analysis of rotavirus genetic material [26].

## Results

**Figure 1:**
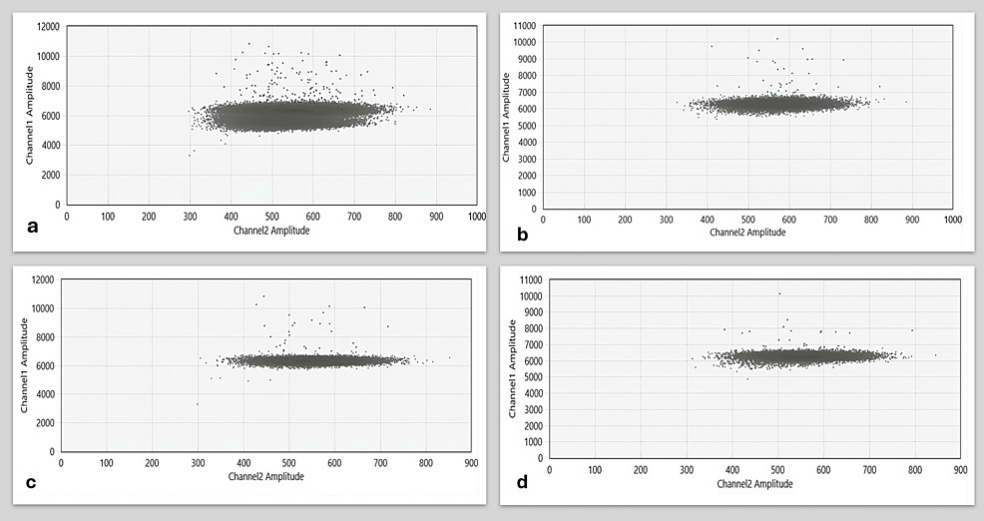
Results of ddPCR. Amplitude 2D analysis images (a-d) of rotavirus-positive samples collected from Chunabhatti WWTPs during April and July 2023 at fortnightly intervals depict rotavirus-positive droplets that are blue in color. ddPCR: droplet digital polymerase chain reaction

**Figure 2:**
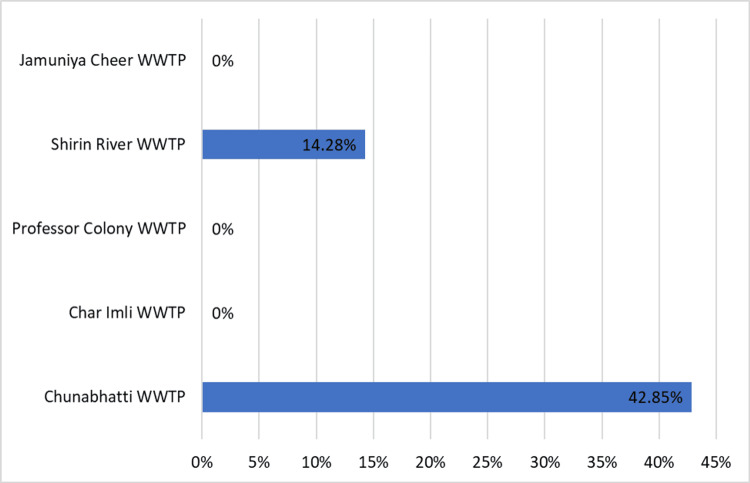
Distribution of Rotavirus in wastewater treatment plants in Bhopal. The X-axis shows the prevalence of Rotavirus in different wastewater treatment plants.

Among 42 samples from diverse WWTPs in Bhopal City, seven exhibited positive rotavirus droplets (Figure 1). Details of the samples from each WWTP, the number testing positive, and the corresponding positivity percentage are shown in Table 1. Notably, six out of 14 samples attached to a hospital at WWTP showed positive results, yielding a 42.85% positivity rate (Figure 2), indicating a high rotavirus occurrence in the treated wastewater. Conversely, no rotavirus evidence was found in samples from three other WWTPs, resulting in a 0% positivity rate; in one other WWTP near the hospital, one out of seven samples tested positive, reflecting a 14.28% positivity rate (Figure 2), suggesting a moderate rotavirus occurrence. Overall, the combined rotavirus positivity rate from all WWTPs was 16.60%, indicating a confirmed prevalence across Bhopal City's WWTPs during the study period.

**Table 1:** Distribution of Rotavirus in wastewater treatment plants in Bhopal City.

S. No.	Name of WWTP	No. of Sample Collected	No. of Rotavirus-Positive Samples	Positivity %
1.	Chunabhatti	14	6	42.85%
2.	Char Imli	7	0	0%
3.	Professor Colony	7	0	0%
4.	Shirin River	7	1	14.2%
5.	Jamuniya Cheer	7	0	0%
	Total	42	7	16.6%

**Figure 3:**
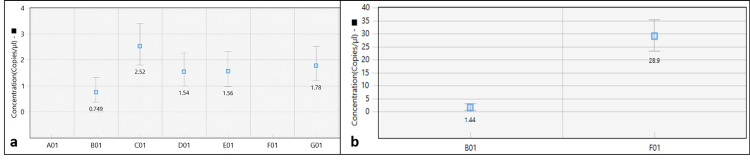
Concentration of Rotavirus in wastewater samples. Figures 3a, 3b represent the concentration of Rotavirus in rotavirus-positive samples. The X-axis in Figures 3a, 3b shows the different samples.

Rotavirus concentrations in positive samples, representing viral loads, were calculated using QuantaSoft software and Poisson statistics. Rotavirus concentrations ranged between 0.75 and 28.9 copies/µL (Figure 3). A sewage water sample from one WWTP associated with a hospital presented the highest concentration of 28.9 copies/µL, indicating elevated viral contamination, whereas a sample from a WWTP nearby this hospital had the lowest concentration of 1.44 copies/µL. ddPCR precision in rotavirus quantification is crucial for gauging risks of viral contamination in wastewater and in understanding their implication for the environment and public health.

## Discussion

This study has been designed to develop a sensitive, specific, and advanced method to investigate the presence of Rotavirus within wastewater samples. Central to this investigation was the assessment of the applicability of ddPCR for the sensitive detection and quantification of Rotavirus. The results obtained from this study provided valuable insights into several important aspects. It shed light on the distribution of Rotavirus across various WWTPs, the concentration of the virus in positive samples, and the potential implications for both environmental integrity and public health.

Among the collected samples, approximately 16.60% exhibited positivity for Rotavirus, indicating a moderate presence of the virus within the Bhopal region. Particularly noteworthy was the Chunnabatti Hospital WWTP, demonstrating the highest positivity rate of 42.85%. This finding suggested a substantial occurrence of Rotavirus within its treated wastewater and was in agreement with the previously published study that ddPCR is useful for the absolute quantification of sewage samples [27]. Conversely, other WWTPs registered a 0% positivity rate. Hospital WWTPs receive influent from patients; therefore, there is a high chance of viral presence. The absence of viruses in other WWTPs might not indicate better treatment; rather, there is a difference in the quality of influent. Our finding potentially points towards the need for effective treatment practices. The water samples from the WWTP showed a 14.28% positivity rate for Rotavirus, suggesting a moderate level of viral presence in the treated wastewater.

The findings of the study underscore the need for intensive monitoring and preventive measures, particularly in WWTPs, as seen with high rotavirus positivity rates. Recent progressions in disinfection techniques include UV irradiation, which targets viral genomic material through the formation of pyrimidine dimers and direct photolysis, effectively damaging the virus [25]. Similarly, viral inactivation by ozone and chlorine-based disinfectants occurs through the oxidation of viral proteins and genetic damage, showcasing their potential in controlling waterborne rotavirus transmission. However, these measures cannot be considered effective for decontaminating viruses from water sources. Therefore, effective management strategies should be formulated to combat viral contamination and protect environmental and public health. The use of ddPCR in the study enabled accurate and sensitive determination of Rotavirus in positive samples [28], allowing insight into the viral load and the extent of contamination. The quantified Rotavirus concentrations in positive samples ranged from 0.75 to 28.9 copies/µL, which is in good agreement with a previous ddPCR assay reported by Racki et al. [28], wherein a rotavirus RNA count of 10 copies/10 µL sewage samples was observed. The higher concentration witnessed in specific samples, such as the one collected from Chunnabatti Hospital, accentuates the potential risks associated with elevated viral loads within wastewater. The precision of ddPCR in quantifying viral concentrations is indispensable for gauging contamination severity and formulating appropriate remediation strategies.

The findings from this study hold important implications for environmental health and public safety. Detection and quantification of Rotavirus in wastewater not only act as good indicators of environmental contamination but also point towards potential health threats [29]. This work suggests that advanced molecular techniques, such as ddPCR, should be incorporated to allow better viral detection, thereby more accurately assessing contamination levels.

The findings suggest that using sophisticated molecular techniques such as ddPCR can improve the detection of viruses, leading to a more precise evaluation of contamination levels. Multiple studies in different countries have demonstrated that monitoring the environment can serve as a valuable means of comprehending the Rotavirus present in a community [30]. As the RV vaccine is being rolled out in numerous countries, it is imperative to explore innovative approaches for detecting and concentrating the virus, particularly in regions that have limited resources. These methods are of utmost importance in conducting environmental surveillance of RV, which is absolutely critical for the triumphant implementation of vaccination programs on a global scale [27].

While revealing significant insights into the presence, distribution, and quantification of Rotavirus within Bhopal's wastewater, the present study is not devoid of limitations. One notable limitation lies in the sampling frequency, which was constrained to weekly intervals. This periodicity might not capture short-term fluctuations in Rotavirus prevalence, potentially underrepresenting temporal dynamics. Furthermore, the study's geographical scope was confined to the Bhopal region, limiting the generalizability of findings to other locales. Additionally, while ddPCR proved invaluable in precise quantification, its application did not delve into genetic diversity or strain variations of Rotavirus. This avenue remains unexplored and presents a promising direction for future research. Untreated sewage needs longer studies to determine the seasonal variation of these viruses [27]. The study also did not consider the impact of environmental factors, such as temperature variations, on Rotavirus persistence. Comprehensive investigations could incorporate these factors to provide a more nuanced understanding. Despite these limitations, this research paves the way for future prospects. Long-term monitoring studies could capture seasonal trends and dynamic changes in rotavirus presence. Adopting advanced sequencing techniques could unravel genetic diversity and potential shifts in viral strains. Furthermore, expanding the study's geographic reach would facilitate a broader understanding of the rotavirus dynamics.

## Conclusions

In conclusion, this study explores the presence of Rotavirus and its quantification in Bhopal’s WWTPs, utilizing ddPCR technology. We found varied rotavirus prevalence across different WWTPs in Bhopal, underscoring the necessity of tailored interventions. Wastewater surveillance and ddPCR can be an early warning system for viral outbreaks. This can help communities prepare, contain the spread, and launch vaccination campaigns efficiently. This research significantly contributes to expanding the knowledge base in health organisations on the practical implications of advanced molecular techniques for water quality management and public health protection, making it a valuable resource for researchers and practitioners alike.
